# Cesarean Section Rate Analysis in a Tertiary Hospital in Portugal According to Robson Ten Group Classification System

**DOI:** 10.1055/s-0040-1712127

**Published:** 2020-06

**Authors:** Sara Vargas, Susana Rego, Nuno Clode

**Affiliations:** 1 Departamento de Ginecologia, Obstetrícia e Medicina da Reprodução, Hospital de Santa Maria, Centro Hospitalar de Lisboa Norte, Lisboa, Portugal

**Keywords:** classification, cesarean section, delivery, classificação, cesarianas, parto

## Abstract

**Objective** The Robson 10 group classification system (RTGCS) is a reproducible, clinically relevant and prospective classification system proposed by the World Health Organization (WHO) as a global standard for assessing, monitoring and comparing cesarean section (CS) rates. The purpose of the present study is to analyze CS rates according to the RTGCS over a 3-year period and to identify the main contributors to this rate.

**Methods** We reviewed data regarding deliveries performed from 2014 up to 2016 in a tertiary hospital in Portugal, and classified all women according to the RTGCS. We analyzed the CS rate in each group.

**Results** We included data from 6,369 deliveries. Groups 1 (*n* = 1,703), 2 (*n* = 1,229) and 3 (*n* = 1,382) represented 67.7% of the obstetric population. The global CS rate was 25% (*n* = 1,594). Groups 1, 2, 5 and 10 were responsible for 74.2% of global CS deliveries.

**Conclusion** As expected, Groups 1, 2, 5 and 10 were the greatest contributors to the overall CS rate. An attempt to increase the number of vaginal deliveries in these groups, especially in Groups 2 and 5, might contribute to the reduction of the CS rate.

## Resumo

**Objetivo** A Classificação de Robson é um instrumento reprodutível, clinicamente relevante e prospectivo proposto pela Organização Mundial de Saúde (OMS) para avaliar, monitorar e comparar as taxas de cesarianas. O objetivo do presente estudo é analisar a taxa de cesarianas ao longo de 3 anos de acordo com a Classificação de Robson e identificar os grupos que contribuíram mais significativamente para essa taxa.

**Métodos** Recolhemos dados relativos aos partos ocorridos entre 2014 e 2016 em um hospital terciário de Portugal e classificamos todas as grávidas de acordo com a Classificação de Robson. Analisamos a taxa de cesarianas em cada grupo.

**Resultados** Incluímos dados relativos a 6,369 partos. Os grupos 1 (*n* = 1,703), 2 (*n* = 1,229) e 3 (*n* = 1,382) representaram 67.7% da população obstétrica. A taxa de cesarianas foi de 25% (*n* = 1,594). Os grupos 1, 2, 5 e 10 contribuíram para 74.2% da taxa de cesarianas.

**Conclusão** Tal como esperado, os grupos 1, 2, 5 e 10 foram os que mais contribuíram para a taxa de cesarianas. Aumentar o número de partos vaginais nestes grupos, principalmente nos grupos 2 e 5, poderá contribuir para a redução da taxa de cesarianas.

## Introduction

Cesarean section (CS) was originally conceived as a life-saving intervention to reduce maternal and fetal mortality and it is nowadays the most commonly performed obstetric procedure.^1^ During the last decades, CS rates have continued to rise worldwide and it became a major public health concern, based on the potential maternal and perinatal risks associated.^2^ ^3^ Several strategies to reduce CS rates have been described in the past few years. Recently, the World Health Organization (WHO) adopted the Robson ten group classification system (RTGCS) as a global standard for assessing, monitoring and comparing CS rates.^4^ This was supported one year later by the International Federation of Gynecology and Obstetrics (FIGO).^5^ This system was presented in 2001 and prospectively classifies women into 10 groups based on 5 characteristics that are routinely documented: parity, onset of labor, fetal presentation, gestational age and number of fetuses (Chart 1).^6^

**Chart 1:** Robson Ten Group Classification System

Group	Description
1	Nulliparous, single cephalic, ≥37 weeks, in spontaneous labor
2	Nulliparous, single cephalic, ≥37 weeks, induced or CS before labor
3	Multiparous (excluding previous CS), single cephalic, ≥37 weeks, in spontaneous labor
4	Multiparous (excluding previous CS), single cephalic, ≥37 weeks, induced or CS before labor
5	Previous CS, single cephalic, ≥37 weeks
6	All nulliparous breeches
7	All multiparous breeches
8	All multiple pregnancies (including previous CS)
9	All abnormal lies (including previous CS)
10	All single cephalic, ≤36 weeks (including previous CS)

Abbreviation: CS, cesarean section.

**Source:** Robson^6^.

Portugal is one of the European countries with the highest CS rates, reaching 32.3% in 2013.^7^ Even though, over the last years, certain Portuguese hospitals have achieved a significant reduction in CS rates only with the implementation of simple measures.^7^ ^8^ ^9^ ^10^ To complement these strategies, in 2015, Portugal also adopted the RTGCS as one of the forms of cesarean classification.^11^

In the present study, we sought to analyze the CS delivery rates in a tertiary public hospital according to the RTGCS over a 3-year period and to identify the main contributors for this rate.

## Methods

Our hospital has ∼ 2,300 deliveries per year and represents a tertiary university/public maternity were no CS is performed based on maternal request. From 2014 up to 2016, we reviewed data from all women who delivered at our institution regarding parity (nulliparous, multiparous, number of previous CSs), onset of labor (spontaneous, induced, prelabor CS), fetal presentation (cephalic, breech, transverse), gestational age (preterm, term) and the number of fetuses (single, multiple) and classified each of them according to the RTGCS. We analyzed CS data concerning the overall CS rate and the contribution of each group to this number. We also examined the size of each group and its individual CS rate. Statistical analysis was performed using the Chi-squared and the Fisher tests. *P* values < 0.05 were considered statistically significant. IBM SPPS Statistics for Windows, Version 19 (IBM Corp., Armonk, NY, USA) was used for the statistical analysis.

## Results

**Fig. 1:**
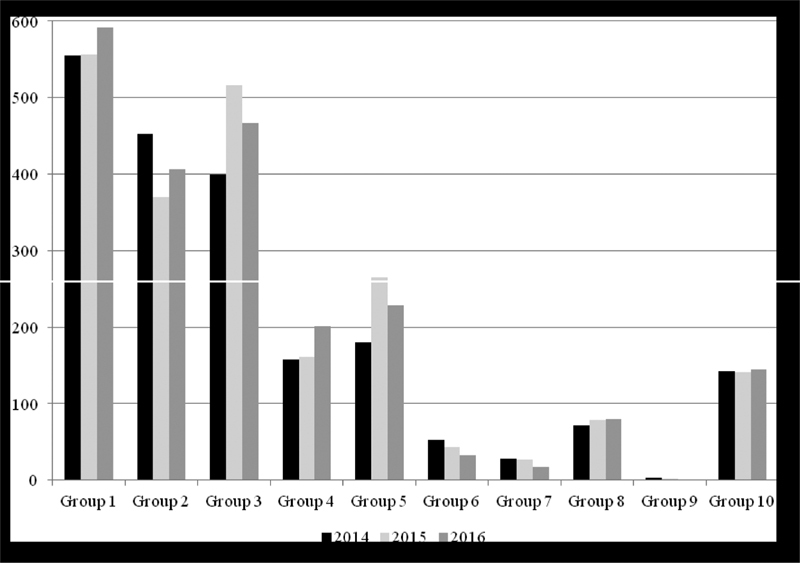
Number of deliveries in each group per year from 2014 to 2016.

During the study period, there were 6,846 (2,193 in 2014; 2,275 in 2015; 2,378 in 2016) deliveries in our department. Data from 6,369 deliveries were analyzed, since 477 cases were excluded due to missing data. The overall CS rate was 25% (*n* = 1,594) with a slight reduction from 26.4% (*n* = 539) in 2014 to 24.3% (*n* = 527) in 2016 (*p* = 0.113). The labor induction rate was 26.7% (*n* = 1,701), and 3.6% (*n* = 229) of all women had multiple gestations. Groups 1, 2 and 3 represented 67.7% (*n* = 4,314) of the obstetric population, and nulliparous with single cephalic full-term pregnancies (Groups 1 and 2) represented almost 50% (*n* = 2,932). A total of 673 women (10.6%) that had a single cephalic full-term pregnancy had also a previous CS (Group 5). Single breech presentations (Groups 6 and 7), twins (Group 8) and single abnormal lies (Group 9) accounted for a minority of deliveries (6.8%, *n* = 435). Preterm cephalic singletons (Group 10) represented 6.7% (*n* = 428) of all deliveries and out of this, 78 (1.2%) had < 33 weeks of gestation. Fig. 1 shows the number of deliveries in each group per year. Although not significant, from 2014 up to 2016 there was an increase in the number of deliveries in Groups 1, 3, 4 and 5, and a reduction in Group 7. There was a significant reduction in the number of deliveries in Groups 2 (*p* = 0.05) and 6 (*p* = 0.018).

Chart 2 presents the CS rate by group and the absolute and relative contribution of each group to the overall CS rate. Cesarean section rates were higher in Groups 9 (single abnormal lie), 6 and 7 (single breech presentation) and 5 (previous CS, single cephalic, ≥ 37 weeks). The great contributors to the overall cesarean rate were Group 2 (nulliparous, single cephalic, ≥ 37 weeks, induced or CS before labor) and Group 5 (previous CS, single cephalic, ≥ 37 weeks) followed by Group 1 (nulliparous, single cephalic, ≥ 37 weeks in spontaneous labor) and Group10 (single cephalic, ≤ 36 weeks). Groups 3 (multiparous without previous CS, single cephalic, ≥ 37 weeks in spontaneous labor), 7 (multiparous, single breech presentation) and 9 (single abnormal lie) had the lowest contribution. Nulliparous women with single cephalic full-term pregnancy (Groups 1 and 2) had a greater impact on the overall CS rate than multiparous women with previous CS with single cephalic full-term pregnancy (Group 5).

**Chart 2:** Cesarean section rates according to Robson ten group classification system and the absolute and relative contribution of each group for the overall cesarean section rate, from 2014 up to 2016

	Number of CS	Number of deliveries	CS rate^a^ (%)	Relative size of the group^b^ (%)	Absolute contribution to the overall CS rate^c^ (%)	Relative contribution to the overall CS rate^d^ (%)
Group 1	171	1703	10 (171/1,703)	26.7 (1,703/6,369)	2.7 (171/6,369)	10.7 (171/1,594)
Group 2	393	1229	32 (393/1,229)	19.3 (1229/6,369)	6.2 (393/6,369)	24.7 (393/1,594)
Group 3	27	1382	2 (27/1,382)	21.7 (1,382/6,369)	0.4 (27/6,369)	1.7 (27/1,594)
Group 4	78	519	15 (78/519)	8.1 (519/6,369)	1.2 (78/6,369)	4.9 (78/1,594)
Group 5	452	673	67.2 (452/673)	10.6 (673/6,369)	7.1 (452/6,369)	28.4 (452/1,594)
Group 6	116	128	90.6 (116/128)	2.0 (128/6,369)	1.8 (116/6,369)	7.3 (116/1,594)
Group 7	56	72	77.8 (56/72)	1.1 (72/6,369)	0.9 (56/6,369)	3.5 (56/1,594)
Group 8	129	229	56.3 (129/229)	3.6 (229/6,369)	2.0 (129/6,369)	8.1 (129/1,594)
Group 9	6	6	100 (6/6)	0.1 (6/6,369)	0.1 (6/6,369)	0.4 (6/1,594)
Group 10	166	428	38.8 (166/428)	6.7 (428/6,369)	2.6 (166/6,369)	10.4 (166/1,594)
TOTAL	1594	6369	25 (1594/6,369)	100 (6369/6,369)	25 (1594/6,369)	100 (1,594/1,594)

Abbreviation: CS, cesarean section.

a % (number of cesarean sections in the group/number of deliveries in the group)

b % (number of deliveries in the group/total number of deliveries)

c % (number of cesarean sections in the group/ total number of deliveries)

d % (number of cesarean sections in the group /total number of cesarean sections)

Chart 3 shows the CS rates from 2014 up to 2016. From 2014 up to 2016, there were no significant reductions in the rate of CS performed in each group. Nevertheless, the rates of CS in each group decreased over time except in groups 9 and 10. Furthermore, there was no change in the main contributors for the overall CS rate.

**Chart 3:** Cesarean section rates according to Robson ten group classification system and the absolute and relative contribution of each group for the overall cesarean section rate in each year, from 2014 up to 2016

	CS rate^a^ (%)			Absolute contribution to the overall CS rate^b^ (%)			Relative contribution to the overall CS rate^c^ (%)		
	2014	2015	2016	2014	2015	2016	2014	2015	2016
Group 1	10.3 (57/555)	10.1 (56/556)	9.8 (58/592)	2.8 (57/2,040)	2.6 (56/2,159)	2.7 (58/2,170)	10.6 (57/539)	10.6 (56/528)	11 (58/527)
Group 2	34 (154/453)	29.5 (109/370)	32 (130/406)	7.6 (154/2,040)	5.1 (109/2,159)	6 (130/2,170)	28.6 (154/539)	20.6 (109/528)	24.7 (130/527)
Group 3	2 (8/399)	1.9 (10/516)	1.9 (9/467)	0.4 (8/,2040)	0.5 (10/2,159)	0.4 (9/2,170)	1.5 (8/539)	1.9 (10/528)	1.7 (9/527)
Group 4	17.2 (27/157)	13 (21/161)	14.9 (30/201)	1.3 (27/2,040)	1 (21/2,159)	1.4 (30/2,170)	5 (27/539)	4 (21/528)	5.7 (30/527)
Group 5	69.4 (125/180)	64.5 (171/265)	68.4 (156/228)	6.1 (125/2,040)	7.9 (171/2,159)	7.2 (156/2,170)	23.2 (125/539)	32.4 (171/528)	29.6 (156/527)
Group 6	94.2 (49/52)	93 (40/43)	81.8 (27/33)	2.4 (49/2,040)	1.9 (40/2,159)	1.2 (27/2,170)	9.1 (49/539)	7.6 (40/528)	5.1 (27/527)
Group 7	85.7 (24/28)	74.1 (20/27)	70.6 (12/17)	1.2 (24/2,040)	0.9 (20/2,159)	0.6 (12/2,170)	4.5 (24/539)	3.8 (20/528)	2.3 (12/527)
Group 8	62 (44/71)	53.9 (42/78)	53.8 (43/80)	2.2 (44/2,040)	2 (42/2,159)	2 (43/2,170)	8.2 (44/539)	8 (42/528)	8.2 (43/527)
Group 9	100 (3/3)	100 (2/2)	100 (1/1)	0.2 (3/2,040)	0.1 (2/2,159)	0.1 (1/2,170)	0.6 (3/539)	0.4 (2/528)	0.2 (1/527)
Group 10	33.8 (48/142)	40.4 (57/141)	42.1 (61/145)	2.4 (48/2,040)	2.6 (57/2,159)	2.8 (61/2,170)	8.9 (48/539)	10.8 (57/528)	11.6 (61/527)
TOTAL	–	–	–	26.4 (539/2,040)	24.5 (528/2,159)	24.3 (527/2,170)	100 (539/539)	100 (528/528)	100 (527/527)

Abbreviation: CS, cesarean section.

a % (number of cesarean sections in the group/number of deliveries in the group)

b % (number of cesarean sections in the group/ total number of deliveries)

c % (number of cesarean sections in the group /total number of cesarean sections)

## Discussion

The overall CS rate in our study was 25%. Despite being less than the CS rates reported by other institutions and countries, it is still higher than the rate between 10 and 15% purposed in 1985 by the WHO.^4^ Although many clinicians consider that such numbers are difficult to achieve, this threshold has been reaffirmed by others, and a recent systematic review confirmed that higher CS rates were not associated with lower mortality.^12^ Nevertheless, in our institution, there was a decrease of the CS rate over time which was better understood after applying the RTGCS.

The RTGCS includes all women in groups that are mutually exclusive, totally inclusive, simple and easy to understand and organize.^12^ It helps to identify which women were being submitted to CS, to define goals regarding each group and to compare results over time.^13^ ^14^ ^15^ ^16^ In our population, Groups 2 and 5 were the greater contributors for the overall CS rate, followed by Groups 1 and 10. Similar findings were reported by other investigators.^2^ ^6^ ^17^ ^18^ ^19^ ^20^ ^21^ Nevertheless, this is not the reality of other institutions, reflecting, once more, different practices and realities.^22^ ^23^

From 2014 up to 2016 there were no significant reductions in the rate of CS performed in each group, but there was a decrease in these rates in all Groups except in Groups 9 and 10. The maintenance of our approach and evaluation might show a significant reduction in the future.

Group 5 (previous CS, single cephalic, ≥ 37 weeks) was the main contributor (28.4%) to the overall CS rate. The fear of a uterine rupture, when a vaginal birth is considered in this population, may reflect this number. Although we have not evaluated the proportion of women with > 1 previous CS and the rate of inductions in this group, the CS rate reported is lower than that described by others.^2^ ^3^ ^16^ ^18^ ^20^ One possible reason for this achievement may be the policy adopted by the Department of using Foley catheter as a mechanical method for cervical priming and labor induction in women with one previous uterine scar which allows vaginal births with no documented increase on maternal or neonatal morbidity.^24^ The number of deliveries in this group has increased over time, reflecting the number of primary CSs and of > 1 previous CS performed in the past.^17^ ^22^ Since in Portugal women may choose the public maternity where they want to deliver, it is possible that the knowledge of the lower CS rate in our institution and particularly in this group might have had influence on this choice.

Nulliparous women with a single cephalic full-term pregnancy (Groups 1 and 2) had a greater impact on the overall CS rate than multiparous women with previous CS with single cephalic full-term pregnancy (Group 5), but they also represent almost half of our population and we know that the rate of CS is higher in nulliparous than in multiparous women. In Portugal, as in other European countries, the natality rate is decreasing with subsequent reduction of the number of multiparous women over time. The ratio between Groups 1 and 2 (< 2:1) reflects a high induction and prelabor CS rate and results in a greater impact on the overall CS rate. Nevertheless, there was a slight reduction in the rates of CS in these groups over time, especially in Group 2, which reflects an attempt to reduce the rate of primary CS performed. The increase in maternal age and morbidities such as obesity, hypertension, diabetes and autoimmune diseases might partially justify the high number of CSs performed.

The number of women with a single cephalic pre-term pregnancy (Group 10) and the high CS rate in this group reflect the characteristics of our department. Many women choose and are followed at our department based on their obstetric and clinical history and mainly because they have a high risk pregnancy. Besides that, we work in a tertiary hospital capable of managing life-threatening maternal and newborn conditions, to where pregnancies are transferred when a preterm delivery is considered. The rates of induction of labor and elective cesareans were not analyzed, but we believe that they might contribute to the high CS rate in this group. Furthermore, the optimal mode of delivery for preterm pregnancies remains controversial, but the CS rate is usually higher than in full-term pregnancies, especially with very low birth weight newborns.^25^

The number of breech deliveries (Groups 6 and 7) in our population was similar to the referred in the literature.^26^ Since the beginning of the present study, there was a significative reduction in the number of deliveries in Group 6 (*p* = 0.018). This may be explained by a reduction of full-term breech pregnancies because we regularly perform external cephalic version before term with a rate of success of 46.9%.^27^ Despite the conclusions of the Term Breech Trial published in 2000, we are making an effort to perform more breech deliveries based on the favorable outcomes documented in the last years contributing to a decrease in the CS rate in this group.^28^ ^29^

In our study, the proportion of multiple pregnancies (Group 8) may be due to the fact that our department is a national reference center for medically assisted procreation. Despite the fact the CS rate in this group is considered low (56.3%) it contributed to 8.1% of the overall CS rate. Even though we did not analyze the proportion of elective and intrapartum CS performed, we believe that a policy of tolerating more prolonged deliveries and a better training for possible complications may lower even more the CS rate in this group.

Multiparous women with single cephalic full-term pregnancy (Groups 3 and 4) represented 30% of all deliveries and, as expected, had a low contribution to the overall CS rate (6.6%), reflecting that CSs are performed based on clinical criteria rather than on a maternal request.

The retrospective analysis of the data collected is a limitation of our study. A longer study period may further confirm the trend in reduction of the CS rate. Missing data may contribute to some bias in the interpretation of the results.

## Conclusion

As expected, nulliparous women with a single cephalic full-term pregnancy that were induced or submitted to an elective CS (Group 2) and women with a previous CS and single cephalic full-term pregnancy (Group 5) were the greatest contributors to the overall CS rate. Also, nulliparous women with a single cephalic full-term pregnancy that went into spontaneous labor (Group 1) and women with single cephalic preterm pregnancy (Group 10) accounted for 21.1% of this number. An attempt to increase the number of vaginal deliveries in these groups, especially in Groups 2 and 5, might contribute to the reduction of the CS rate.
